# The Senior Sex Education Experience Study: Qualitative Data From Developing an Adult Sex Education Program

**DOI:** 10.1093/geroni/igaa057.1615

**Published:** 2020-12-16

**Authors:** Christina Pierpaoli-Parker

**Affiliations:** University of Alabama at Birmingham, Vestavia Hills, Alabama, United States

## Abstract

Older adults (OAs) account for an unprecedented proportion of STDs but receive limited sex education. We present preliminary data from the SEXEE study developing a sex education intervention for OAs 50+. The sample consisted of 17 OAs, ages 53 to 77, (M = 65, SD = 7.6; 64.7% female; 94.1% Caucasian) and seven primary care physicians, geriatricians, and other specialists (e.g. gynecologists), sampled purposively. Physicians completed a semi-structured interview to describe their experiences discussing sexual health with OAs, identify barriers to those discussions, and elicit recommendations for an educational curriculum. OAs participated in three separate focus groups to determine their interest in and suggestions for the intervention. Qualitative data underwent thematic coding separately by two researchers, with a third researcher resolving any discrepancy. One physician (14%) reported routinely assessing adults’ sexual health; others only in the context of a specific presenting concern (e.g. ED). Though the physicians considered sexuality important component of QoL, many reported barriers to assessment and treatment, including insufficient time, training, and knowledge; concerns about personal and patient discomfort; and patient complexity. Of the OAs interviewed, 15 (88%) endorsed high interest in attending a sex education program. The most commonly recommended educational topics among physicians and OAs included: sexual changes with aging and management strategies; STDs and risk factors; tools for enlarging the sexual repertoire, myths about late life-sexuality; masturbation, and dating. Findings extend previous observations about clinical barriers to sexual health discussions and provide new insights for developing a sex ed intervention for this population.

